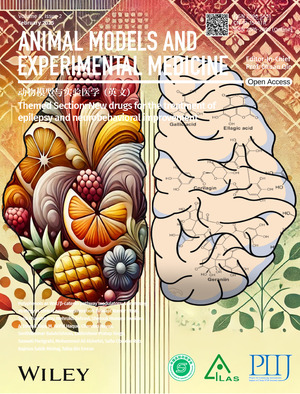# Cover Picture

**DOI:** 10.1002/ame2.70008

**Published:** 2025-02-28

## Abstract

This cover image is based on the article "Polyphenols as Wnt/β‐catenin pathway modulators: A promising strategy in clinical neurodegeneration" (https://doi.org/10.1002/ame2.12525) reported by Biswajit Kumar Utpal, Sajib Chandra Roy, Mehrukh Zehravi, Sherouk Hussein Sweilam, A. Dinesh Raja, M. Akiful Haque, Chandan Nayak, Senthilkumar Balakrishnan, Laliteshwar Pratap Singh, Saswati Panigrahi, Mohammed Ali Alshehri, Safia Obaidur Rab, Najmus Sakib Minhaj, Talha Bin Emran. The cover design encapsulates the study's main focus: "Exploring the Neuroprotective Benefits of Phytochemicals Extracted from Indigenous Edible Fruits in Bangladesh". This is achieved by integrating several symbolic aspects in order to visually depict the core idea and theme of the paper and its emphasis on neuroscience, natural compounds, and local biodiversity. (1). Brain as the central symbol: A prominent, stylized brain illustration serves as the focal point representing the study's neuroprotective aspect. Chemical structures, including Gallic acid, Ellagic acid, Corilagin, and Geraniin, encircle the brain, illustrating the phytochemicals under investigation for their potential advantages. (2). Fruits symbolizing Phytochemical Sources: The abstract geometric patterns subtly suggest fruits and their visual placement making them embedded within the brain directly symbolizing a strong connection between the Indigenous fruits and their neuroprotective properties. Moreover, the vibrant color scheme and the ordered representation further emphasize the phytochemical advantages connected with the fruits and their beneficial impact on brain health. (3). Cultural and Regional Symbolism: The striking background of the cover image takes inspiration from the nakshi kantha motifs which are traditional embroidery designs originating from Bangladesh and, therefore, integration of this aspect honors the cultural context associated with the research.